# *Potamopyrgus antipodarum* as a potential defender against swimmer’s itch in European recreational water bodies—experimental study

**DOI:** 10.7717/peerj.5045

**Published:** 2018-06-25

**Authors:** Anna Marszewska, Anna Cichy, Jana Bulantová, Petr Horák, Elżbieta Żbikowska

**Affiliations:** 1 Department of Invertebrate Zoology, Faculty of Biology and Environment Protection, Nicolaus Copernicus University of Torun, Toruń, Poland; 2 Department of Parasitology, Faculty of Science, Charles University, Prague, Czech Republic

**Keywords:** *Potamopyrgus antipodarum*, *Radix balthica*, *Trichobilharzia regenti*, Miracidia, “Decoy effect”

## Abstract

Swimmer’s itch is a re-emerging human disease caused by bird schistosome cercariae, which can infect bathing or working people in water bodies. Even if cercariae fail after penetrating the human skin, they can cause dangerous symptoms in atypical mammal hosts. One of the natural methods to reduce the presence of cercariae in the environment could lie in the introduction of non–host snail species to the ecosystem, which is known as the “dilution” or “decoy” effect. The caenogastropod *Potamopyrgus antipodarum*—an alien in Europe—could be a good candidate against swimmer’s itch because of its apparent resistance to invasion by European bird schistosome species and its high population density. As a pilot study on this topic, we have carried out a laboratory experiment on how *P. antipodarum* influences the infestation of the intermediate host *Radix balthica* (a native lymnaeid) by the bird schistosome *Trichobilharzia regenti*. We found that the co–exposure of 200 *P. antipodarum* individuals per one *R. balthica* to the *T. regenti* miracidia under experimental conditions makes the infestation ineffective. Our results show that a non–host snail population has the potential to interfere with the transmission of a trematode via suitable snail hosts.

## Introduction

Biodiversity loss and disease emergence have become two of the most challenging issues confronting science and society (Johnson et al., 2009). Different authors indicate the strong correlation between parasite success in ecosystems and the biodiversity of ecological communities (Johnson et al., 2012; Lagrue & Poulin, 2015). Mitchell et al. (2003), Begon (2008), Allan et al. (2009) as well as many others underlined that rapid loss of populations significantly increase disease emergence. Studies concerning the causal relationship between biodiversity and disease emergence in an environment are focused on testing the “dilution effect”, which parasitologists refer to as a “decoy-effect” hypothesis (Combes & Moné, 1987; Johnson & Thieltges, 2010). According to these authors, the “decoy effect” mechanisms, observed in the case of high biodiversity of ecological communities, concern: (i) degeneration of invasive parasite stages penetrating the non-target host, (ii) exhausting of these stages by trying to penetrate the non-target host and (iii) stimulation of defense mechanisms of the non-target host against invasive stages of parasite. Regardless of the mechanism, the non-target host becomes the dead-end host, that is the real factor reducing the parasitic disease emergence (Mehlhorn, 2008).

One of the re-emerging worldwide medical problems connected to parasites of complex life cycle is cercarial dermatitis, also known as swimmer’s itch (Cort, 1936; Hunter et al., 1949; Jarcho & Van Burkalow, 1952; Macy, 1952; Hoeffler, 1974; Leedom & Short, 1981; Eklu-Natey et al., 1985; Blankespoor & Reimink, 1991; Loken, Spencer & Granath Jr, 1995; Pilz, Eisels & Disko, 1995; Lindblade, 1998; Kolářová, Skírnisson & Horák, 1999; Horák et al., 2015). Marszewska et al. (2016) observed this medical problem in many bathing localities in Polish Lowland Lakes during the last two years. The dermatitis appears as an itchy, lumpy rash on the skin that persists for several weeks (Żbikowska, Wójcik & Grygon-Franckiewicz, 2002). The skin lesions resemble the early stage of chickenpox, and are a result of penetration by cercariae of bird schistosomes (Żbikowska, 2003). Normally, cercariae of bird schistosomes develop inside the host snail for six to seven weeks (Amen & Meuleman, 1992). Cercariae then abandon the mollusk, swim in the water environment seeking to penetrate the skin of an avian final host; once in the skin they transform to schistosomulae, then they migrate through the blood or nervous pathway, mature, and reproduce sexually (Soldánová et al., 2013). If a human becomes the accidental target of a cercariae attack, an allergic skin reaction may follow, but the worms do not mature in humans (Kolářová, Horák & Skírnisson, 2010; Horák et al., 2015).

The current increase in the number of swimmer’s itch cases in temperate climate might be a consequence of both: (i) climate change accompanied by the extension of the period of active vegetation in freshwater ecosystems, linked with abundant populations of host snails releasing bird schistosome cercariae, and (ii) people spending more time in recreational activities (Angilletta Jr, 2006; Rempfer et al., 2010). Biomass of cercariae of the bird schistosome *Trichobilharzia szidati* can even reach 4.65 tons per year for a small eutrophic reservoir (Soldánová, Selbach & Sures, 2016).

The above factors limit safe water recreation (Chamot, Toscani & Rougemont, 1998; Lévesque et al., 2002; Farahnak & Essalat, 2003; Skírnisson & Kolárová, 2005; Jouet et al., 2008). Efforts to reduce human cercarial dermatitis have been made by using some trematode species or by lowering the density of first intermediate host snail populations; however, such efforts were not always successful (Chapter 1; Loker & Hofkin, 2015). The mechanical removal of potential intermediate hosts of bird schistosomes brings only limited positive effects (Dubois, 2003), and the use of molluscicides, however successful for a short period, has a clear limitation (see as review: King & Bertsch, 2015) or even a negative impact on local fauna (McCullough, 1992).

The increasing number of cases of human cercarial dermatitis together with our knowledge on migration of bird schistosomes in mammalian hosts (Horák & Kolářová, 2001; Horák et al., 2008; Horák et al., 2015) foster research on natural methods that may decrease the risk. As for human schistosomes, biological control has been tested in some areas (see review: Pointier, David & Jarne, 2011) and promising results have been obtained for the use of applied alien or even invasive snail species for reduction of parasite prevalence in the snail hosts. The “decoy effect” described by Combes & Moné (1987) can be another mode of resolving the problem. Combes & Moné (1987) indicate that *Schistosoma mansoni* miracidia can fail to actively penetrate non-host snails*.* We suspect that the same variant of biological control can be useful in the case of bird schistosomes, especially when using the planned alien species for human cercarial dermatitis control—*Potamopyrgus antipodarum*, which has been present in European waters for years (Boycott, 1936; Walter, 1980; Dorgelo, 1987; Ponder, 1988; Simoes, 1988; Hinz, Boeters & Guenther, 1994; Berg et al., 1997; Carlsson, 2000; Wagner, 2000; Mouthon & Dubois, 2001). The presence of this New Zealand native species has been recorded in several European countries (Gérard & Le Lannic, 2003; Zettler & Richard, 2004; Sousa, Guilhermino & Antunes, 2005; Alonso, 2006; Lewin & Smolinski, 2006; Soler, 2006; Cianfanelli, Lori & Bodon, 2007; Múrria, Bonada & Prat, 2008; Son, Nabozhenko & Shokhin, 2008; Zieritz & Waringer, 2008; Radea, Louvrou & Economou-Amilli, 2008; Arle & Wagner, 2013), but only a few reports have given it the status of invasive species (Brzeziński & Kołodziejczyk, 2001; Gaino et al., 2008; Thomsen et al., 2009). *P. antipodarum* spreads easily thanks to its wide tolerance to environmental factors and its parthenogenetic reproduction, so a population can start from a single female. In some non–native regions even up to six generations per year can develop (Piechocki & Wawrzyniak-Wydrowska, 2016). *P. antipodarum* can create populations with densities reaching thousands of individuals per square meter under favorable conditions (Richards, Cazier & Lester, 2001; Hall Jr, Tank & Dybdahl, 2003), but densities may undergo a drastic collapse in a few months (Extence, 1981; Moffitt & James, 2012) or in a longer term (Moore et al., 2012; Gérard, Hervé & Hechinger, 2017). These top-down and bottom-up changes in invaded ecosystems can be extremely temporally dynamic and connected to environmental factors (Moore et al., 2012). Among the reasons for a collapse, an impact of acquired parasites was postulated. Even if parasites cannot complete the life cycle in *P. antipodarum* due to host–parasite incompatibility (Żbikowski & Żbikowska, 2009), the penetration of miracidia or cercariae through the tegument can be devastating for snails. The expansion of *P. antipodarum* in European waters and scarce cases of its stable association with a trematode species (Gérard & Le Lannic, 2003; Morley, 2008; Gérard, Hervé & Hechinger, 2017; Żbikowska & Nowak, 2009) resulted in the hypothesis that the introduction of *P. antipodarum* to the European bathing localities may help eliminate the risk of dermatitis in a safe way.

Our pilot laboratory experiments aimed at evaluating the potential impact of *P. antipodarum* on the effectiveness of *T. regenti* (an avian schistosome) miracidia to infect the natural, native host snail *Radix balthica*.

## Materials and Methods

### Snails

*Radix balthica* (Linnaeus, 1758) (Pulmonata: Basommatophora: Lymnaeidae) is one of the most common pond snails in Poland (Piechocki & Wawrzyniak-Wydrowska, 2016). Based on external morphology, these snails are similar to *R. labiata* (Rossmassler, 1835). Therefore, the species-level taxonomy within the *Radix* genus was verified on the basis of anatomical features of the reproductive system (Schniebs et al., 2011). *R. balthica* is the intermediate host for many digenean species, such as bird schistosomes, including *T. regenti* (Horák, Kolářová & Dvořák, 1998; Cichy, Faltynkova & Żbikowska, 2011). In the experiment, 40 *R. balthica* individuals with shell height of 8–10 mm (mean size: 9.0 ± 0.1) and shell width of 4–6 mm (mean size: 5.1 ± 0.1) (very susceptible to parasitic invasion) were used. All *R. balthica* individuals came from laboratory breeding cultures of the Department of Invertebrate Zoology at *Nicolaus Copernicus University* in Toruń, Poland.

*Potamopyrgus antipodarum* (Gray, 1843) (Caenogastropoda, Hydrobioidea, Tateidae) is a mud snail species introduced from New Zealand to Europe in the mid 1850s (Hubendick, 1950). In Poland it was first found in Lake Trląg (Urbański, 1938). Nowadays it is common in Pomerania, Greater Poland, Masurian Lakeland and Upper Silesia (Cichy, Faltynkova & Żbikowska, 2011). In the experiment, parthenogenetic females with shell height of 4 mm (most prevalent during summer season in Poland) were used. The snails were collected from Sosno Lake (53°20′15″N, 19°20′55″E) in May 2016.

### Bird schistosome

*Trichobilharzia regenti* (Schistosomatidae, Bilharziellinae) was described by Horák, Kolářová & Dvořák (1998). As for the maintenance of parasites in the laboratory, the intermediate host snails of *Radix lagotis* were kept in aquaria with sponge filters, fed on lettuce leaves, and repeatedly collected and placed in glass beakers to stimulate release of cercariae after lighting. The definitive hosts, ducks (*Anas platyrhynchos* f. dom.), were kept in cages approved for this purpose (accreditation no. 13060/2014-MZE-17214). Their infection with cercariae was performed as described by Meuleman, Huyer & Mooij (1984). After 22 days, the ducks were sacrificed by decapitation to obtain eggs with developing miracidia, and adult trematodes living in the nasal mucosa. The maintenance care and sacrificing of experimental animals was carried out in accordance with European Directive 2010/63/EU and Czech law (246/1992 and 359/2012) for biomedical research involving animals. Experiments have been performed under legal consent of the Expert Committee of the Section of Biology, Faculty of Science, Charles University, Prague, Czech Republic, and the Ministry of Education, Youth and Sports of the Czech Republic under ref. no. MSMT-31114/2013-9.

Four ducks in the patent period (22 days post infection) were sacrificed, and their heads immediately (within 10 h) transported to the Polish laboratory at the temperature of 8°C. In the laboratory nasal conchae were removed from the duck beaks and torn apart in Petri dish with conditioned tap water to release eggs and hatched miracidia, which were then placed in a dark flask with conditioned tap water at 20°C. The flask was placed under artificial light. After a few minutes, hatched miracidia were concentrated under illuminated water surface. The larvae were then individually collected with a micropipette.

### Experiment I: the infection of *P. antipodarum* snails with miracidia of *T. regenti*

*P. antipodarum* individuals were experimentally infected in Petri dishes (50 mm in diameter) with conditioned tap water at 20°C. In the experiment two variants were applied—one miracidium per one snail, and five miracidia per one snail. The experiment was performed in 25 replicates. The time of exposure was 20 h, and was adjusted to the duration of the miracidia life span (Horák et al., 2015). Then snails were carefully placed into beakers with conditioned tap water at 20°C, and water in Petri dishes was checked under a stereomicroscope for presence of living or dead miracidia.

Twice a week, the water in incubation beakers was changed, and the *P. antipodarum* individuals were fed. Every day the activity of snails was tested. Dead individuals were immediately checked for parasite infestation. After 60 days all surviving snails were killed and autopsied.

### Experiment II: the infection of *R. balthica* in the presence of *P. antipodarum* with miracidia of *T. regenti*

During this experiment snails were exposed to miracidia in Petri dishes (50 mm in diameter) filled with conditioned tap water at 20°C. In the experiment, three variants of non–host snail density were applied: 50, 100 and 200 individuals of *P. antipodarum* per one individual of *R. balthica*. The size of experimental *P. antipodarum* groups was determined according to the ratio of number specimens of both snail species per square meter in different Polish water bodies (Żbikowski & Żbikowska, 2009; Strzelec, Krodkiewska & Królczyk, 2014). *R. balthica* were placed individually in the central part of the dish, whereas *P. antipodarum* specimens were arranged around them. For each dish with snails, three newly hatched miracidia of *T. regenti* were added, according to a common laboratory procedure (Lichtenbergová et al., 2011). The dishes were covered and placed in the incubator (SANYO, Osaka, Japan) at 20°C and natural photoperiod for 24 h (adjusting the time to the maximum survival of larvae). The experiment was performed in 10 replicates. The three control groups of snails constituted (i) separately placed individual of *R. balthica* with three miracidia (without *P. antipodarum*), and (ii) separately placed individual of *P. antipodarum* with three miracidia (without *R. balthica*)—both in 10 replicates. The additional, third control consisted of only one Petri dish with fifty *P. antipodarum* snails incubated together with fifty miracidia (Table 1). After 24 h, the control snails were rinsed with water and placed separately in beakers with conditioned tap water at 20°C. Similarly to experiment I, the water was changed and the snails were fed twice a week. Every day their activity was observed. Dead individuals were immediately checked for parasite infestation. After 60 days all remaining (living) snails were killed and autopsied.

**Table 1:** The exposure of host and/or non-host snails on *Trichobilharzia regenti* miracidia at 20°C—Experiment II.

Number of snails exposed to parasitic larvae^a^	Number of miracidia	Number of replicates	Experimental condition
1 *R. balthica* + 50 *P. antipodarum*	3	10	Experimental
1 *R. balthica* + 100 *P. antipodarum*	3	10	Experimental
1 *R. balthica* + 200 *P. antipodarum*	3	10	Experimental
1 *R. balthica*	3	10	Control
1 *P. antipodarum*	3	10	Control
50 *P. antipodarum*	50	1	Control

**Notes.**

a Animals were placed in Petri dishes of 50 mm diameter.

### Statistical analysis

The prevalence of *T. regenti* in both snail species populations was counted as percent of specimens of *R. balthica* or *P. antipodarum* with bird schistosome larvae (sporocysts and/or cercariae). In order to verify if the density of *P. antipodarum* accompanying *R. balthica* can affect *T. regenti* miracidia infectivity, logistic regression was used, with the *Potamopyrgus antipodarum* abundance as a predictor variable and infection status of *Radix balthica* as a dependent variable. Snail life time since exposure to miracidia, expressed in number of days, was analyzed by one–way ANOVA, followed by post–hoc Tukey test. Significant differences in the survival rates between infected and uninfected *R. balthica* were tested using Mann–Whitney *U* test.

## Results

**Figure 1:**
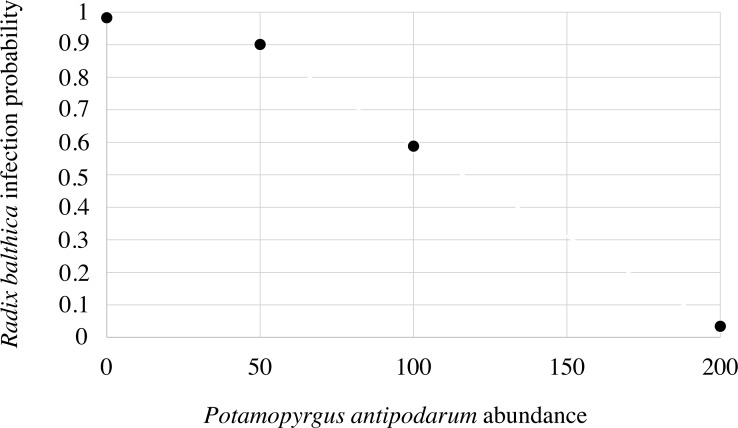
Infection probability of *Radix balthica* predicted by the logistic regression model on the basis of the abundance of *Potamopyrgus antipodarum*.

None of the *P. antipodarum* individuals exposed to *T. regenti* miracidia in Experiment I or Experiment II were found to be infected. During Experiment I, after 20 h of incubation we did not find parasitic larvae in the water of the Petri dishes, where *P. antipodarum* snails were individually exposed to three miracidia. Also, no patent infection (with fully developed cercariae) was found in *R. balthica* specimens experimentally exposed to *T. regenti* miracidia. Inside the infected snails only sporocysts or sporocysts with immature cercariae were noticed. The effective infestation of *R. balthica* by *T. regenti* (Table S1) depended on the number of accompanying *P. antipodarum* individuals during exposure to miracidia (Table S2). Abundance of *Potamopyrgus antipodarum* significantly decreased the probability of infection of *Radix balthica* (logistic regression: Wald statistic = 9.5, *df* = 1, *p* = 0.002) (Fig. 1). Almost all control *R. balthica* individuals (90%), and all *R. balthica* snails co-incubated with 50 specimens of *P. antipodarum* exposed to miracidia had parasite sporocysts. The infestation of *R. balthica* was completely ineffective in the density combination of 200 *P. antipodarum* per one *R. balthica* specimen (*p* < 0.0001). When 100 *P. antipodarum* individuals were co–exposed to miracidia, up to 40% *R. balthica* snails were non–infected, however, the difference was not statistically significant if compared with the *P. antipodarum*—absent control (*p* = 0.0867).

**Figure 2:**
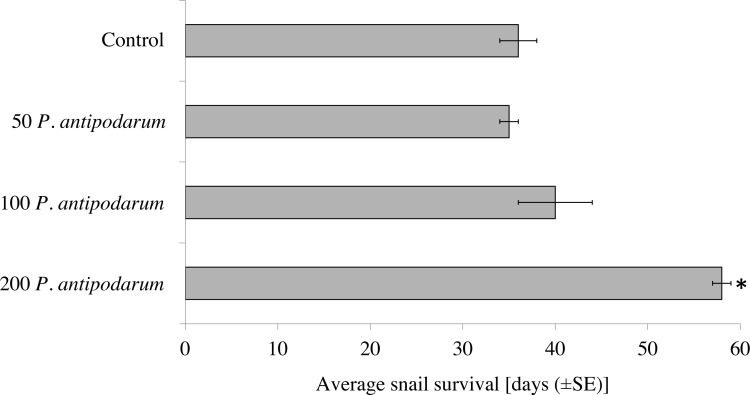
Survival of *Radix balthica* exposed to *Trichobilharzia regenti* miracidia in the presence of different number of *Potamopyrgus antipodarum* specimens. (*) the Mann–Whitney *U* test: *p* < 0.001.

Experimental conditions had an impact on the life span of snails. All *P. antipodarum* individuals survived until the end of the experiment (60 days), whereas the life span for *R. balthica* varied (Table S3). The average survival of *R. balthica* ranged from 35 to 58 days, and depended on the presence of non–host *P. antipodarum* snails during exposure to miracidia (one–way ANOVA F_3,36_ = 16.85, *p* < 0.001)). Post–hoc tests indicated that the longest survival rate occurred in the case of *R. balthica* exposed to miracidia in the presence of 200 individuals of *P. antipodarum* (Fig. 2). Additionally, the Mann–Whitney *U* test (*p* < 0.001) indicated that infected *R. balthica* lived shorter than non–infected ones regardless of experimental condition (avg. 34 ±1 and 57 ±1 days respectively) (Table S4, Fig. 2). Infected *R. balthica* survived for 18–40 days (range) after exposure to miracidia, while most non-infected *R. balthica* lived until the end of the 60 day experiment.

## Discussion

Our study indicates that *T. regenti* larvae cannot use *P. antipodarum* as an intermediate host succesfully. This is not surprising because all known *Trichobilharzia* species use only Lymnaeidae and Physidae as intermediate hosts (Horák et al., 2015). On the other hand, the lack of miracidia in water after 20 h exposure to snails is extremely interesting. It could be the effect of parasitic larvae entering the snail shell or even possible attachment to non-host *P. antipodarum* body. Sapp & Loker (2000) observed miracidia which tended to adhere to incompatible snails, but these larvae could not develop inside a non-host mollusk. It should be noted that according to King, Jokela & Lively (2011) trematodes have only one chance when they attach to a snail body—succesful infection or death. Combes & Moné (1987) described the impact of non-target hosts on parasite success as a “decoy effect” and suggested the “decoy effect” as potentially useful in schistosomiasis control. Unfortunately, the protocol of our experiment did not allow us to track the fate of *T. regenti* miracidia, because the snails were stored in the incubator during the exposure period. Observations after the end of exposure revealed the lack of invasive larvae on Petri dishes, which could be the result of them being swallowed by snails or the effect of the degeneration of the unsuccessful larvae that died during the experiment. The only certainty is that the presence of non–host snails (*P. antipodarum*) of bird schistosome (*T. regenti*) in the neighborhood of native host (*R. balthica*) can affect the parasite transmission success of miracidia. The hatched larvae respond to different environmental stimuli, such as light or gravity, and various chemical compounds released by potential host species (Hertel et al., 2006). Smyth & Halton (1983) when using the choice–chamber to study miracidial chemo–orientation indicated that nearly half of the tested larvae were attracted by chemical attractants other than those released by their specific host snail. *P. antipodarum* individuals co–exposed to miracidia may have successfully disturbed the access of parasitic larvae to the specific host. However, the lack of data on the chemical composition of *P. antipodarum* mucus does not allow a clear conclusion that the lack of miracidia in water after 20 h exposure to individuals of this species during Experiment I could support our hypothesis.

According to Sullivan & Yeung (2011), miracidia that were experimentally injected into snails were encapsulated inside incompatible hosts, but survived and developed in the compatible ones only. The result shows that real recognition of the intruder by the immune system of the host depends on the internal milieu of the snail. This fact allows us to understand why the imprecise identification of the host by miracidia does not result in snail—Digenea compatibility (Combes & Moné, 1987). We suggest that the probable lack of precision in *T. regenti* miracidia orientation could be used for the biological control of this trematode invasion in the environment.

The introduction of an alien snail species into the environment, even to protect people against parasites, may raise doubts concerning long-term consequences of manipulation in the environment: (i) the influence on populations of native snail species, and (ii) the danger of a new parasite–snail association. As for the first consequence, data on the displacement of native European snail species by *P. antipodarum* seem to be exaggerated. Some statistical analysis has shown the coincidence between the appearance of *P. antipodarum* in water bodies and a drop in Simpson’s diversity index. Such an analysis was presented by Strzelec, Spyra & Krodkiewska (2006) who used number of individuals as currency in the Simpson’s diversity index. It should be emphasized that the large numbers of the small *P. antipodarum* could easily drive down the Simpson index (or any other abundance-based diversity index) if numbers are used as currency. In our opinion the biomass would be more appropriate currency in such analysis.

The threat of new snail-parasite association seems to be more serious collateral damage to planned manipulation in the environment (Morley, 2008). However, it must be emphasized that *P. antipodarum* already occurs in European waters, and the possible introduction into recreational waters would only slightly increase its range (Städler et al., 2005). From New Zealand, where this mud snail plays the role of intermediate host for many avian parasite species, there are no reports of infection of *P. antipodarum* with *Trichobilharzia* species (Hechinger, 2012), even if *Trichobilharzia quequedulae* was noted in birds of the Southern Hemisphere (Ebbs et al., 2016). The facts above indicate potentially safe use of *P. antipodarum* against swimmer’s itch.

The absence of patent infection in *R. balthica* individuals after an experimental exposure is also of interest. Huňová et al. (2012) underlined that the intramolluscan development of *T. regenti* needs several weeks. In our experiments, none of the successfully infected *R. balthica* lived longer than 40 days. The increased mortality of snails experimentally infected with trematodes is widely known (Muñoz Antoli et al., 2007; Kalinda, Chimbari & Mukaratirwa, 2017), especially when juvenile snails are exposed to miracidia. In our experiments, the *R. balthica* snails that remained uninfected after exposure to *T. regenti* lived longer than the infected ones (Fig. 2). Many of them survived until the end of the 60 day experiment. As the limiting factor for parasite invasion of *R. balthica* seems to be the presence of *P. antipodarum* individuals, it can be concluded that the presence of non–host snails during exposure to miracidia indirectly increased the survival of *R. balthica* hosts. Although the results do not show clear evidence of the non–invasive nature of *P. antipodarum*, they may suggest an additional, indirect effect of this snail species on native malacofauna in new areas. Our results highlight the additional aspect of the influence of alien snail species on native malacofauna. Ecologists emphasize the direct changes caused by newcomers (Riley, Dybdahl & Hall Jr, 2008). In our opinion, especially in the case of research on freshwater snails, the aspect of their association with trematodes should be taken into account in analyses concerning the impact of alien species introduction.

## Conclusion

The data represent a pilot study that precedes a wide–planned series of field and laboratory studies focused on the influence of alien molluscan species, namely *P. antipodarum*, on possible reduction of swimmer’s itch in European recreational water bodies. Our experimental work demonstrates that a high population density of *P. antipodarum* lowers the transmission of bird schistosomes miracidia to suitable snail hosts such as *R. balthica*. Further research will focus on the potential ability of *P. antipodarum* to limit native gastropod infections in natural conditions.

##  Supplemental Information

10.7717/peerj.5045/supp-1 Table S1 Effectiveness of parasite invasion to *Radix balthica* in the presence of *Potamopyrgus antipodarum*

10.7717/peerj.5045/supp-2 Table S2 The database for effectiveness of parasite invasion to *R. balthica* at the presence of *Potamopyrgus antipodarum* (results of Fisher Exact Test)

10.7717/peerj.5045/supp-3 Table S3 Survival of *Radix balthica* infected with *Trichobilharzia regenti* miracidia in the presence of *Potamopyrgus antipodarum*

10.7717/peerj.5045/supp-4 Table S4 Survival of infected and non–infected *Radix balthica*

## Additional Information and Declarations

### Data Availability

The following information was supplied regarding data availability: The raw data are supplied in the Supplemental Files.
